# AffectMachine-Classical: a novel system for generating affective classical music

**DOI:** 10.3389/fpsyg.2023.1158172

**Published:** 2023-06-06

**Authors:** Kat R. Agres, Adyasha Dash, Phoebe Chua

**Affiliations:** ^1^Yong Siew Toh Conservatory of Music, National University of Singapore, Singapore, Singapore; ^2^Centre for Music and Health, National University of Singapore, Singapore, Singapore; ^3^Augmented Human Lab, DISA, National University of Singapore, Singapore, Singapore

**Keywords:** automatic music generation system, algorithmic composition, music MedTech, emotion regulation, listener validation study, affective computing

## Abstract

This work introduces a new music generation system, called AffectMachine-Classical, that is capable of generating affective Classic music in real-time. AffectMachine was designed to be incorporated into biofeedback systems (such as brain-computer-interfaces) to help users become aware of, and ultimately mediate, their own dynamic affective states. That is, this system was developed for music-based MedTech to support real-time emotion self-regulation in users. We provide an overview of the rule-based, probabilistic system architecture, describing the main aspects of the system and how they are novel. We then present the results of a listener study that was conducted to validate the ability of the system to reliably convey target emotions to listeners. The findings indicate that AffectMachine-Classical is very effective in communicating various levels of Arousal (*R*^2^ = 0.96) to listeners, and is also quite convincing in terms of Valence (*R*^2^ = 0.90). Future work will embed AffectMachine-Classical into biofeedback systems, to leverage the efficacy of the affective music for emotional wellbeing in listeners.

## 1. Introduction

There is now overwhelming evidence that music supports health and well-being in various ways, from motivating physical activity, to promoting mental health and fostering social connection (MacDonald et al., 2013; Fancourt and Finn, 2019). Music is particularly effective for supporting and mediating emotion states. Indeed, one of the primary reasons people report listening to music is to change or enhance their emotions (Thayer et al., 1994; Lonsdale and North, 2011; Saarikallio, 2012). Given the affordances of music to support health and wellbeing, as well as advances in machine learning and computational techniques, there has recently been a call to action to compose music with the use of computational technologies for healthcare applications (Agres et al., 2021). Compared to the use of human-composed, pre-recorded music, which spans many genres and emotions but is fixed and difficult to adjust in real-time, generative music composition systems are able to support real-time interactivity—they are able to flexibly manipulate musical features almost instantaneously according to the listener's current neural or physiological state, or given their real-time input and preferences. These systems therefore show promise in delivering personalized, cost-effective (and free of copyright), non-invasive, non-pharmaceutical methods for helping individuals improve their emotion states. Given the global mental health crisis (e.g, nearly 20% of adults in the USA live with a mental illness, or 52.9 million Americans in 2020^1^), music medtech systems are projected to be extremely valuable tools for supporting emotional wellness, and mental health more broadly.

More generally, in an age where computational systems are now being used extensively to generate impressive natural language and visual art, such as the technologies available through OpenAI,^2^ it is no surprise that there has been a recent surge of interest in the development of automatic music generation systems (AMGSs; also known as algorithmic composition systems). Like human music composition and improvisation, AMGSs generally aim to create harmonic, timbral, and rhythmic sequences in an organized, musically-coherent fashion. This area, which sits at the intersection of computing, music theory/composition, and computational creativity, is relatively nascent, however, compared to the computational creation of visual art. This work aims to not only chip away at this gap, but offer a new automatic music generation system—AffectMachine-Classical—that is capable of producing controllable *affective* music. AffectMachine-Classical offers an effective and flexible means of conveying emotions in real-time, and the system has been developed to be embedded into biofeedback systems such as brain-computer-interfaces (see for example Ehrlich et al., 2019), making it a potentially powerful tool for therapeutic applications such as emotion and mood regulation in listeners, augmentation of physical activity during rehabilitation, as well as commercial use cases such as soundtrack design and providing silent videos with novel music free of copyright.

### 1.1. Related work

A review of automatic music generation is out of the scope of this article (for a review and summary of the state-of-the-art, see Herremans et al., 2017; Carnovalini and Rodà, 2020; Dash and Agres, 2023), however we will briefly summarize the main approaches to automatic music generation. Previous approaches to developing music generation systems largely fall into two categories: learning-based methods, and rule-based methods. While there has been a recent trend toward learning-based approaches, they present several challenges for affective music generation. First, ecological (or realistic) music pieces typically exhibit hierarchical, long-term structure, as well as polyphony. For example, a melodic phrase may extend over multiple measures of music, and involve several different instruments or voices. Further, music typically has an overall form that allows for musical and stylistic coherence throughout the piece. As such, a generative model must be able to capture harmonic, rhythmic and temporal structure, as well as the interdependency between voices (Dong et al., 2018). Second, learning-based approaches require large music datasets with emotion labels for training, a resource that is still scarce in the community, although we note that acoustic models that are able to link musical excerpts directly to natural language descriptions are beginning to emerge (Huang et al., 2022), and may be a promising direction for future work. Style transfer models have had success as an alternative to models capable of generating novel affective music from scratch—for example, Ramirez et al. (2015) used machine learning models to apply appropriate expressive transformations on the timing and loudness of pre-composed input musical pieces based on desired arousal and valence. In addition, Williams et al. (2017) used affective feature mappings to transform seed material generated by a neural network trained on short musical excerpts, and Hu et al. (2020) used convolutional neural networks to extract stylistic features from therapeutic music pieces and incorporate them into user-selected songs. Despite these promising applications, style transfer models and similar approaches are subject to several important limitations. For example, although leveraging pre-composed music greatly simplifies the challenge of producing affective music, the pre-existing music chosen is subject to copyright. Recent progress in conditional music generation from text has resulted in models that are able to generate high-fidelity music based on natural language descriptions (Agostinelli et al., 2023), which may potentially sidestep copyright issues. However, style transfer models and generative models are not yet able to support flexible and continuous generation for real-time interactivity, which is essential in biofeedback systems or any other systems meant to compose music in real-time to mediate the user's affective states.

In comparison to learning-based approaches, rule-based approaches rely on hand-designed functions to map affective signals to musical parameters. As such, they are able to sidestep the challenges associated with learning-based approaches by building in knowledge of how affective states map to musical parameters, as well as typical expectations regarding harmonic, rhythmic, and temporal structure. Additionally, the design of rule-based affective music generation systems benefits from an extensive body of theoretical and empirical work going back almost a century that investigates how different aspects of musical structure contribute to emotional expression (Gabrielsson and Lindström, 2010). For example, the system described in Wallis et al. (2011) was primarily informed by Gabrielsson and Lindström (2001), and generates novel music algorithmically by mapping seven musical parameters (e.g., note density, musical mode) to either valence or arousal in the most continuous possible way. Even though several salient musical parameters such as tempo, voice leading, and voice spacing, were not mapped for simplicity, the system was sufficient for participants to hear corresponding changes in the emotion of the music when changes were applied to the valence and arousal parameter settings. Similarly, the adaptive music engine described in Gungormusler et al. (2015) manipulates musical parameters including tempo, articulation, and timbre based on empirical validation of music-emotion structural rules carried out by Livingstone and Brown (2005). Most recently, Ehrlich et al. (2019) developed a system that loops over a I-IV-V-I harmonic progression, and modifies the musical mode, tempo, rhythmic roughness (a measure of the amount of variation in note lengths within a measure), overall pitch, and relative loudness of subsequent notes based on the desired level of valence and arousal. Their listening study confirmed a high correspondence between the system's arousal and valence settings and the emotions listeners perceived (Ehrlich et al., 2019).

Compared to existing rule-based systems, AffectMachine is more sophisticated, by taking into account traditional features, such as tempo, rhythmic roughness/note density, mode, etc., as well as additional features such as voice leading and a fine-grained mapping between valence/arousal and musical features such as the chord progression. In addition, because our system is capable of producing music in real-time based on given arousal and valence, it has a flexibility not exhibited by most other music generation systems.

### 1.2. Emotion perception in music

Music listening is often a rich emotional and cognitive experience (Altenmüller and Schlaug, 2012), and numerous studies have explored the relationship between music and emotional expression. For example empirical studies have been carried out to better understand both the emotions that can be expressed through music (e.g., Gabrielsson and Juslin, 2003), as well as the musical factors that contribute to perceived emotional expression (e.g., Gabrielsson and Lindström, 2010). Research has shown that various musical cues, such as tempo, mode, dynamics, pitch range, rhythm, and articulation, can influence the perceived emotion in music (Gabrielsson and Juslin, 2003; Schubert, 2004; Juslin and Västfjäll, 2008; Juslin and Sloboda, 2013). For example, studies have found that fast tempos are associated with positive emotions such as joy and excitement, while slow tempos are associated with negative emotions such as sadness and melancholy (Juslin and Laukka, 2003). Similarly, major modes are generally associated with positive emotions, while minor modes are associated with negative emotions (Juslin and Laukka, 2003), although this can depend on musical enculturation (Swaminathan and Schellenberg, 2015). Other musical cues, such as dynamics/loudness and pitch range, can also influence the perceived emotion in music. For instance, loudness has been found to correlate strongly with perceived and induced arousal, while high pitch ranges are associated with excitement and low pitch ranges with sadness (Balkwill and Thompson, 1999; Swaminathan and Schellenberg, 2015). Overall, these findings suggest that musical features play a crucial role in influencing perceived emotion in music. The connection between musical features and emotion has also led to a surge of research in Music Information retrieval (MIR) which aims to identify the high-level emotions of music from its low-level features (see, for example, Yang et al., 2018), an area often referred to as music emotion recognition.

Studies have found that listeners tend to exhibit agreement in their judgment of the general emotions expressed by a piece of music, and that these judgments are only marginally affected by demographic factors such as musical training, age, and gender (Juslin and Laukka, 2004), although differences in emotion perception have been emerging in recent work examining the impact of factors such as age and musical training (Cohrdes et al., 2020; Koh et al., 2023). In addition, music is often unable to reliably communicate finely differentiated emotions (Juslin, 1997). Sloboda (2004) offers an explanation for this phenomenon, suggesting that music is to a large extent abstract and ambiguous, and while it may be able to suggest varying levels of energy or resemble certain gestures and actions, these emotional contours are often fleshed out in a subjective manner.

Other recent studies have explored the neural mechanisms underlying emotional responses to music, with a particular focus on the role of the brain's reward system. For example, Salimpoor et al. (2015) describes how listening to music activates the brain's reward system, leading to the release of dopamine and other neurotransmitters associated with pleasure and reward. This suggests that our emotional responses to music are not simply a matter of subjective experience, but are also rooted in the underlying biology of the brain, e.g., dopamine is released in concert with prediction mechanisms in the brain during music listening (Huron, 2008; Salimpoor et al., 2015; Ferreri et al., 2019). Overall, these and other recent studies continue to deepen our understanding of the complex relationship between music and emotion, and suggest that systems able to flexibly manipulate musical features have great potential for emotion-focused well-being applications such as affective music generation systems.

Taken together, the literature suggests that (i) to a large extent, music can be systematically modified to express desired emotions, and that (ii) the effectiveness of affective music generation systems should be fairly robust across listeners.

### 1.3. AffectMachine-Classical

The current music generation system, AffectMachine-Classical, uses a probabilistic, rule-based approach to generate affective classical music in real time. The system was developed with the help of a classically-trained composer finishing his studies at a major Conservatory of Music, and the system's generated music generally aims to follow the stylistic conventions of Western tonal classical music^3^.

Various approaches have been used to measure and describe the affective qualities of musical stimuli, ranging from widely used measures such as Russell (1980)'s circumplex model and the Geneva Emotional Music Scale (GEMS; Zentner et al., 2008) to bespoke methods developed for specific studies (e.g., Costa et al., 2000; Lindström, 2006). Following much of the existing work on affective music systems (e.g., Wallis et al., 2011; Ehrlich et al., 2019), we opted to represent emotion in AffectMachine using the circumplex model, in which emotions can be understood as points within a two-dimensional space. The first dimension is arousal, which captures the intensity, energy, or “activation” of the emotion, while the second is valence, which captures the degree of pleasantness. For example, excitement is associated with high arousal and high valence, while contentment would be associated with low arousal and high valence. The circumplex model has several advantages over alternative measures of emotion. Firstly, to provide accurate and fine-grained feedback to a user about his or her emotional state, music generated by AffectMachine should ideally vary smoothly over the entire space of emotions, making continuous models of emotion such as the circumplex model a natural choice over categorical models of emotion such as GEMS. Secondly, allowing musical features to change gradually over time could help lend the music a more natural sound. Finally, the generalizability of the circumplex model also enables us to make use of previous research which may have used less common measures of emotion, by interpreting their results in terms of arousal and valence.

AffectMachine provides a model that is able to fluidly generate affective music in real time, either based on manually-input or predetermined arousal and valence values (e.g., as a sort of affective playlist for emotion mediation, or trajectory through emotion space), or based on the real-time feedback or physiological state of the user (e.g., EEG activity captured from the user and mapped to arousal and valence). In this way, AffectMachine offers a flexible yet powerful way to sonify (real-time) emotion states, and to influence the emotion states of the listener. The system may be used for health and wellness applications, such as generating affective playlists for emotion mediation. Further, AffectMachine may also be integrated into Brain-Computer Interface (BCIs) devices, or other systems capable of providing biofeedback, to assist the user in achieving a desired emotion state through neuro/biofeedback and affective music listening.

The main contributions of this work are: (1) the design of a novel rule-based affective music generation system to compose non-monotonic classical music, and (2) validation of the proposed system for expressing different emotions through a listener study. In the next section of this paper, we describe the features of AffectMachine-Classical (Section 2). We then describe the listener study and discuss the findings and implications of our results (Section 3), before providing our general conclusions and suggested future directions (Section 4).

## 2. AffectMachine-Classical system description

In this section, we describe the parameters and design of our novel affective music generation system, AffectMachine-Classical, which produces affective music in a classical style. AffectMachine was developed to be embedded in a BCI or neurofeedback system, to both generate emotion-inducing music in real-time, and to allow for neural or physiological signals (such as EEG) to *drive* the music generation system. That is, the system was developed to both induce emotion in listeners, and provide users with real-time feedback on their current emotional state, in which the generated music is a reflection (or sonification) of the listeners' emotion state (when AffectMachine is embedded in a BCI or neurofeedback system). In the present paper, we remove AffectMachine from any embedded, interactive contexts (e.g., BCI), and examine the standalone AffectMachine, focusing on the efficacy of AffectMachine for generating music that conveys the intended emotion.

The automatic music generation system was developed in Python, and takes a sequence of arousal and valence states as input and encodes a corresponding sequence of harmonic, rhythmic, and timbral parameters in the form of a MIDI event stream as output. The MIDI event stream is then sent to a digital audio workstation (DAW) over virtual MIDI buses to be translated into sound. For the present version of AffectMachine, we use the Ableton DAW for its wide selection of instruments and its ability to support live multi-track recording. Arousal and valence are continuous values within the range [0, 1] that can either be sampled from sensors (such as EEG) or manually provided. All musical parameters are updated each bar in accordance with the current arousal and valence values.

Developing a rule-based affective music generation system requires first identifying a set of musical parameters and affective states, then designing functions that map parameter values to target states. For this reason, the harmonic, rhythmic, and timbral parameters were selected based on previous work establishing their influence on musical expression of emotions, and developed in collaboration with conservatory students formally trained in music composition.

In the subsections below, we present the details of the AffectMachine-Classical system.

### 2.1. Harmonic parameters

Previous rule-based music generation systems have controlled the mode parameter by choosing a fixed harmonic progression (e.g., I-IV-V-I) and in a few cases, by varying the musical mode from which the chords are drawn (e.g., each musical mode was mapped to a certain level of valence), with Lydian typically identified as the mode that expresses the highest valence, and Locrian or Phrygian as the mode that expresses the lowest valence, as per Schmuckler (1989). A simpler, and much more common, version of this logic is to switch between the major and minor modes.

In the AMG system, we introduce a completely novel way of controlling mode by using a bespoke probabilistic chord progression matrix inspired by the theme and variation form found in (human-composed) classical music. The music loops through an 8-bar theme with fixed chord functions for each bar, but the specific chords used, as well as their probabilities, are determined by the target level of valence desired. To our knowledge, this approach has never before been implemented in a computational music generation system. The chord set available for each level of valence was based on previous empirical work, as well as the musical insights from conservatory students formally trained in music composition. Previous empirical work has established that valence tends to be positively related to the major mode, and negatively related to the presence of dissonance (e.g., diminished and augmented intervals; Costa et al., 2004; Costa and Nese, 2020). Generally, the chords progressions in our system exhibit greater dissonance with higher probability as valence decreases. Arousal had no influence on the chord progression selected.

Unlike previous systems which are constrained to a specific harmonic progression, the AMG system is extremely flexible—the only constraint being that the music has to progress through the 8-bar theme. (Note that the majority of human-composed music also adheres to a repeating X-bar structure). This novel approach is therefore beneficial by allowing a greater range of musical possibilities (and “interestingness” of the composition). At the same time, the music is able to achieve greater coherence of musical structure than what is commonly found in machine learning-based approaches by using chord substitutions in an 8-bar theme to express the desired level of valence, and by ending each iteration of the theme with a cadence.

To craft the 8-bar theme, the valence range was divided into 10 regions, with one probabilistic chord progression composed for each region to match the intended level of valence. For example, at higher levels of valence, the chord progressions are composed in the major mode as it is typically associated with expressions of positive valence. As valence decreases, the likelihood of chords with greater tension or dissonance (such as those with diminished or minor intervals) increases. For a given bar (e.g., 1–8) and level of valence (e.g., 0–1), there are a set of possible chords, each with a particular probability of occurrence from 0.1 to 0.8. At any given bar and valence level, there are typically multiple chords (between one and five) to choose from.

### 2.2. Pitch characteristics of voices

#### 2.2.1. Voice leading

Voice leading refers to the art of creating perceptually independent musical lines (e.g., tenor line, soprano line, etc.) that combine to form a coherent piece (Huron, 2001), and is a steadfast component of the majority of human-composed polyphonic music. Despite the importance of voice-leading, automatic generation of polyphonic music with multiple voices or tracks is a challenge that research is only just beginning to address, primarily with learning-based generative methods (e.g., Dong et al., 2018), and many of these systems either fail to address voice leading altogether or use highly simplified versions of voice leading.

In the AffectMachine system, we implement a novel rule-based music generation system that draws on both traditional rules of voice leading as well as heuristics used by human musicians, to create pieces that exhibit perceptually independent musical lines with nontrivial complexity and variability. (Note that in our system, we utilize and refer to voices, not in the strict sense of counterpoint, but similar to the use of voices in a string quartet, where one voice or instrument is capable of playing a chord.) By mapping these rules to differing levels of arousal and valence, we also provide more cues for listeners to identify the emotion being conveyed by the music, and enable finer-grained control over the mapping between affective states and musical parameters. This is an extremely important aspect and benefit of our approach.

AffectMachine-Classical was developed to generate music with four parts or voices. The four voices/algorithms we employ were selected to fill out the acoustic space from low bass frequencies to the higher soprano range. While the instruments do not map strictly to the counterpoint definition of voices (e.g., with independent bass, tenor, alto, and soprano lines), they do span the frequency spectrum from low to high, and work together to convey a cohesive melody and harmony. The bass voice is carried by the string section, and always plays the root note of the current chord. The principal melody is played by the soprano voice, which is carried by the clarinet and doubled at higher valence settings by the marimba. Both inner voices are carried by the piano, with the tenor voice playing a full chord voicing in the middle register, and the alto voice providing harmonic accompaniment by means of single notes adhering to voice leading principles (the details are described below). Instrumentation is explained in more detail in the section on timbral parameters.

While there are numerous principles that govern voice leading, or the creation of perceptually independent parts (Huron, 2001), we select several straightforward rules that provide sufficient melodic diversity while minimizing unpleasant or artificial-sounding melodic lines. The three parts that are determined through voice leading logic are the tenor, alto, and soprano voices. For the principal melody, our primary goal was to avoid unexpected dissonance. Hence, the note sequence is a randomly selected sequence of chord tones. For the tenor voice, which plays the full chord voicing of the chord progression, we follow the heuristic outlined in Wallis et al. (2011)—that pianists tend to voice new chords in a manner that is as similar as possible to the previous chord, in terms of interval and placement on the keyboard. We calculate dissimilarity between two notesets (*N*, *N*′) as per Equation (1) and select the least dissimilar chord voicing to be played the first inner voice.


(1) $$\begin{array}{l} dissimiliarity=\underset{i}{\sum }\underset{j}{\sum }|N_{i}-N_{j}^{\prime }|\forall i\in N,\forall j\in N^{\prime } \end{array}$$

The alto voice is monophonic, playing one note at a time according to a step motion rule, where the initial note is a randomly selected chord tone. This rule states that if the next note in the *melody* is of a different pitch, the pitch motion of the alto voice should be by diatonic step (e.g., move up or down the diatonic scale). These rules are encoded in the form of transition matrices. There are four possible states: −1, indicating a diatonic step down the scale; 1, indicating a diatonic step up the scale; 0, indicating no pitch motion; and CT, indicating a jump to a randomly selected chord tone (CT). The arousal range was divided into two equal regions, with one matrix composed for each region to generate appropriate melodies for each level of arousal. The transition matrices were developed such that at higher levels of arousal, melodies are more likely to consist of scale patterns, mitigating the risk of the music being too dissonant or unpleasant due to the increased tempo and note density. Note that our system does not directly avoid parallel fifths/octaves (due to the complexity of the system and the presence of many features), but because this is a probabilistic system, movements of fifths in multiple voices at the same time are relatively rare.

#### 2.2.2. Pitch register

Research in the psychology of music has associated pitch height and pitch register with emotional expression for almost a century (Hevner, 1937); yet pitch height is often not explicitly incorporated into automatic music generation systems. Higher pitches generally tend to be associated with positively-valenced emotions such as excitement and serenity (Collier and Hubbard, 1998), while lower pitches tend to be associated with negatively-valenced emotions such as sadness.

In AffectMachine-Classical, the pitch register of the lowest voice is consistent (at C3). For the remaining voices, the pitch register can vary within a permissible range determined by the current valence level.

To implement changes in pitch register, we again divided the valence range into ten equally spaced regions and tuned the lower and upper bounds of allowable pitches by ear. Both the lower and upper bounds of the range of permissible pitches increase gradually as valence increases. The range of permissible pitches starts at [C1, C5] in the lowest valence region, and gradually moves to [G3, C6] in the highest valence region.

### 2.3. Time and rhythm parameters

#### 2.3.1. Rhythm

In most automatic generation approaches, the rhythmic content of the music is either fixed (e.g., a repeating pattern or a pre-composed rhythm template is used), or the temporal duration of notes (the rhythmic content) is based on a machine-learning generative process that affords little musical cohesion. This tends to either make the music sound extremely repetitive, or rather incoherent and unpleasant for most listeners.

To surmount this issue, the different voices/parts/tracks in AffectMachine-Classical use different rhythmic logic, e.g., one voice uses probabilistic rhythms while another uses composed rhythms. In this way, our new approach finds a nice and aesthetically-pleasant balance between composed and probabilistic elements.

As mentioned above, AffectMachine-Classical was developed to generate music using four parts or voices. The bass voice (string section) and first tenor voice (piano) employ a fixed rhythmic pattern—they are both played on the first beat of each bar. For the soprano voice (clarinet and marimba), we divided the arousal range into three regions: low (Arousal < 0.4), moderate (0.4 ≥ Arousal < 0.75) and high (Arousal > 0.75). Much like the implementation of mode, for a given bar (e.g., 1–8) and arousal region, there is a set of two possible rhythmic patterns or “licks” with equal probability of occurrence. The rhythmic pattern is represented in code as a list of binary values indicating whether each beat (subdivision) is associated with a note activation.

Finally, for the alto voice (piano), we incorporate rhythmic roughness, which is a measure of how irregular the rhythm of a piece of music is. Music with smooth, regular rhythms are typically perceived as higher in valence. In AffectMachine-Classical, we use note density as a proxy for rhythmic roughness (Wallis et al., 2011). As arousal increases, roughness decreases, and note density increases. When roughness is 0, each bar is populated with eight notes of equal length. However, this often results in overly dense-sounding output, because tempo is also high at higher levels of arousal. Hence, we limit the lowest roughness to 0.3.

#### 2.3.2. Tempo

Tempo, or beats per minute, determines how quickly the notes of each bar are played. Alternatively, tempo can be thought of as a measure of note duration—the faster the tempo, the shorter the note duration. In AffectMachine-Classical, tempo is determined by a simple linear relationship with arousal, and ranges from 60 bpm at Arousal = 0–200 bpm at Arousal = 1.

### 2.4. Timbral and loudness parameters

Two parameters contributed to variations in timbre: (i) the instrumentation of AffectMachine-Classical, and (ii) the velocity of notes, which refers to the force with which a note is played.

#### 2.4.1. Velocity range

Similar to the algorithmic composition system developed by Williams et al. (2017), we mapped coordinates with higher arousal to brighter and harder timbres that were created by increasing MIDI key velocity. In MIDI, velocity is measured on a scale from 0 to 127. In our system, the range of permissible MIDI key velocities is [40, 70] at Arousal = 0, and the lower and upper bounds of the range increase linearly with arousal to [85, 115] at Arousal = 1. A uniform distribution over the range is used to determine the velocity for each bar.


(2) $$\begin{array}{l} Velocity=unif40+aro*45,70+aro*45 \end{array}$$

#### 2.4.2. Velocity variation

Patterns of velocity variation have affective consequences. For example, research has found that large changes in velocity (loudness) suggest fear, while small variations convey happiness and pleasantness (Scherer and Oshinsky, 1977; Krumhansl, 1997; Juslin and Laukka, 2001; Gabrielsson and Lindström, 2010). Further, rapid changes in velocity may be associated with playfulness or fear (Krumhansl, 1997).

In our experimentation with the system, we found that frequent changes in velocity tend to result in unpleasantly disjointed, artificial-sounding music, and we therefore attempt to limit large, rapid (e.g., unexpected-sounding) variations in velocity. Furthermore, changes in velocity become more frequent as tempo (which is linearly related to arousal, as per Section 2.3.2) increases. Therefore, to strike a balance between enabling sufficient variation in velocity, and incorporating those variations in as natural a way as possible, we limited the maximum change in velocity allowable within each bar. The variation in velocity is dependent on the arousal level and bar of the progression. Specifically, we set an overall minimum and maximum loudness level, and the allowed deviation becomes smaller as arousal decreases. The magnitude of variation in velocity is random, within the allowable range (which is set for each bar), and there are no changes in velocity within each bar.

#### 2.4.3. Instrumentation

Four virtual instruments were employed in the system (piano, a string section, clarinet, and marimba), and used to convey a classical musical style. As mentioned previously, the lowest voice is conveyed by the string section, while both inner voices are carried by the piano. The principal melody is placed in the uppermost voice, which is played by the clarinet. The marimba is used to double over the clarinet at high levels of valence (Valence ≥ 0.8) due to its cheerful-sounding timbre (and because, during experimentation with the system, marimba was found to nicely complement the timbre of the clarinet, which could sound slightly shrill at higher pitch heights). After all other harmonic, rhythmic, and timbral parameters have been determined, instrument samples in the DAW (Ableton) are used to generate the final output audio.

## 3. AffectMachine-Classical listener study

### 3.1. Method

A listening study was conducted in order to validate the efficacy of AffectMachine-Classical for generating affective music. We first used our system to generate brief musical examples from different points around the arousal-valence space of the circumplex model (Russell, 1980). Listeners then provided arousal and valence ratings for each of these excerpts to examine whether the target emotion (in terms of arousal and valence) was indeed perceived as intended by listeners.

#### 3.1.1. Participants

The listening study was conducted with 26 healthy participants (average age = 22 yrs, SD = 4 yrs) including 11 male and 15 female participants. Twelve of the 26 participants reported having prior musical training. All the participants were given verbal and written instructions about the listening study prior to providing their written consent. The study was approved by the Institutional Review Board (IRB) of the National University of Singapore (NUS).

#### 3.1.2. Stimuli

AffectMachine-Classical was designed to compose affective music that can span the entire valence-arousal plane. For the validation study, musical stimuli were generated from 13 different points around the valence and arousal plane. These were meant to represent different emotional states around the space, and covered the corners, middle of each quadrant, and the neutral middle point of the space. The points are: {valence, arousal} = [{0,0}; {0,0.5}; {0,1}; {0.25;0.25}; {0.25,0.75}; {0.5,0}; {0.5,0.5}; {0.5,1}; {0.75,0.25}; {0.75,0.75}; {1,0}; {1,0.5}; {1,1}]. There is a precedent in the literature for selecting these points in the arousal-valence plane for the validation of a music generation system (Ehrlich et al., 2019).

To account for the probabilistic nature of the system, three different musical stimuli were generated from each of the thirteen points, resulting in a total of 39 musical excerpts. This mitigates the risk that artifacts in any particular stimulus might bias listener ratings, for more robust results. The average duration of the music stimuli is 23.6 s. The stimuli were composed based on either an 8- or 16-bar progression to allow the music to reach a cadence. Note that because AffectMachine was designed to generate music continuously and flexibly based on the listener's physiological state or real-time arousal and valence values, the music does not always reach a full cadence at the end of an 8-bar sequence (e.g., sometimes the tonic/cadence is only reached at the beginning of the subsequent 8-bar sequence). In the present case, we are not testing the ability of the music to have well-formed cadences *per se*, but to convey a target emotion. That is, the examples do not necessarily end with a musical cadence; rather, they are excerpts from what could be an infinitely-long musical creation. Therefore, while generating stimuli with a fixed duration is possible, this often results in stimuli that end abruptly, which might influence a listener's emotional response to the stimuli. Sixteen bars were used for stimuli with a fast tempo (e.g., high arousal excerpts), as 8 bars produced too brief a time duration for these excerpts. All musical stimuli were presented to each participant in randomized order to avoid order effects across participants. The music stimuli used in this validation study are available online at: https://katagres.com/AffectMachineClassical_stimuli.

#### 3.1.3. Experimental protocol

The experiment was conducted one participant at a time in a quiet room with minimal auditory and visual distractions. The experimenter first provided verbal and written instructions about the experiment, and then the participant provided written, informed consent to participate in the study. During the listening study, the participant sat in front of a computer and listened to the music stimuli over headphones, with the sound level adjusted to a comfortable listening volume.

Before the listening task, the participant was asked to complete a demographic questionnaire which included questions about his/her age, prior musical training, ethnicity, etc. Subsequently, the participant rated his/her current emotional state.

The music listening study began with two practice trials, followed by the 39 experimental trials in randomized order. After listening to each stimulus, the participant was asked to indicate the *perceived* emotion of the stimulus (that is, the emotions they felt that the music conveyed) on a visual 9-point scale known as the Self-Assessment Manikin (SAM; Bradley and Lang, 1994). These ratings were collected for both arousal and valence. Briefly, valence refers to the degree of the pleasantness of the emotion, while arousal refers to the activation or energy level of the emotion. The SAM scale ranged from “very unpleasant” (1) to “extremely pleasant” (9) for valence, and from “calm” (1) to “excited” (9) for arousal. Participants were allowed to take as long as they required to make these ratings, but were only permitted to listen to each musical stimulus once. The total duration of the experiment was ~40 min, and participants were compensated with $6 SGD (equivalent to $4.50 USD) for their time.

### 3.2. Results and discussion

In order to evaluate the efficacy of the music generation system, we analyzed the user ratings collected during the music listening study. We aimed to investigate (1) whether the music generated by the system is able to express the desired level of valence and arousal to the listeners, and (2) whether perceived valence and arousal are dependent on the listeners' prior musical training/knowledge. In this regard, we present our results in two subsections: (1) arousal and valence ratings, and (2) the impact of prior musical training on emotion ratings. We do not consider demographic factors such as age and ethnicity for further analysis due to the limited sample size.

As is commonly found in listener studies of emotion in music, we observed that the average valence and arousal ratings varied across listeners. This variance is often attributed to individual differences in musical preferences and training, and the listeners' demographic and cultural profile (Koh et al., 2023). In order to mitigate the differences across listeners, we normalized the perceptual ratings from each user (see Equations 3 and 4 below). Here, *Max*_*Valence*_ refers to the maximum possible valence rating (i.e., 9), and *Min*_*valence*_ refers to the minimum possible valence rating (i.e., 1). The same *Max* and *Min* values apply to Arousal. The normalized valence and normalized arousal ratings, ranging between 0 and 1, are used for further analysis. In the remainder of the article, the normalized valence and normalized arousal ratings will be referred to as valence and arousal ratings, respectively.


(3) $$\begin{array}{l} Normalized_{Valence}=\frac{Rated_{Valence}}{\left(Max_{Valence}-Min_{Valence}\right)} \end{array}$$

(4) $$\begin{array}{l} Normalized_{Arousal}=\frac{Rated_{Arousal}}{\left(Max_{Arousal}-Min_{Arousal}\right)} \end{array}$$

#### 3.2.1. Arousal and valence ratings

**Figure 1:**
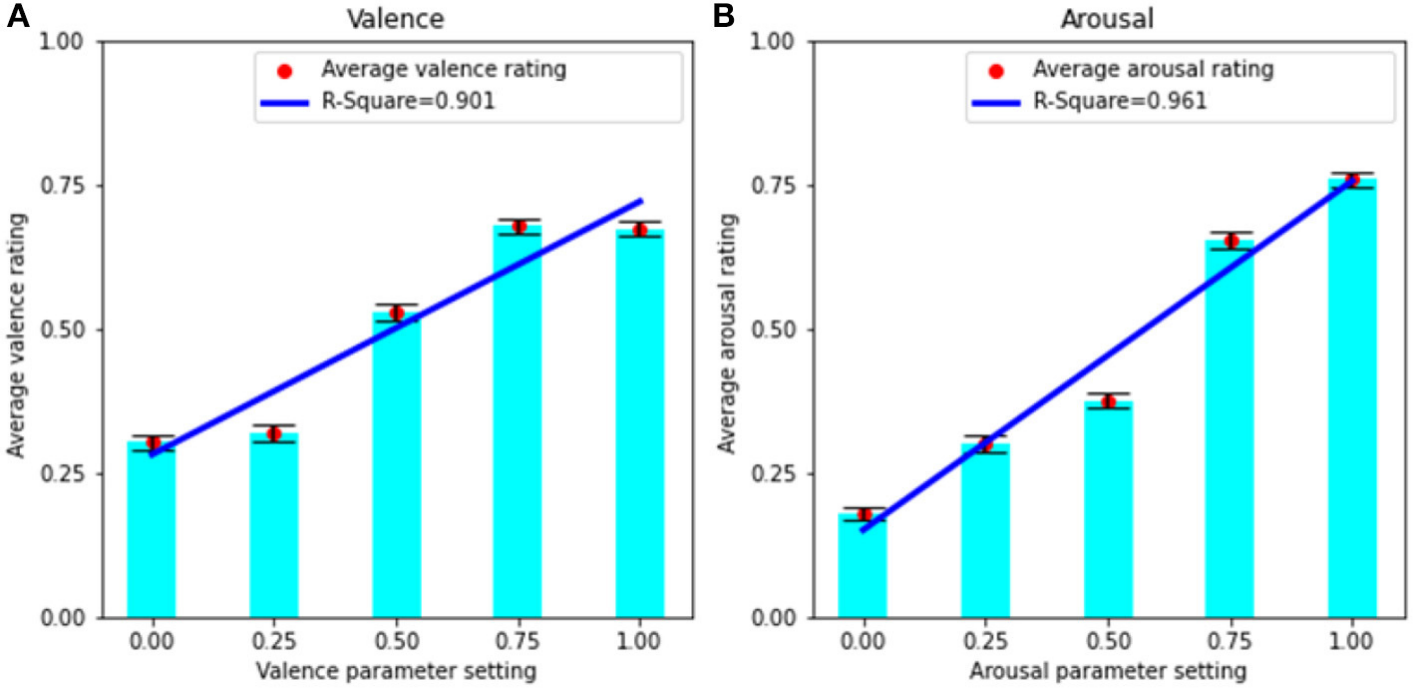
**(A)** Linear regression between parameter settings and average valence ratings. **(B)** Linear regression between parameter settings and average arousal ratings. Error bars display standard error.

To investigate whether AffectMachine is able to accurately express the intended emotion through music, we compared participants' averaged (normalized) emotion ratings for the musical stimuli with the valence or arousal parameter settings used during the music generation process. For example, the averaged valence ratings for all stimuli generated with the parameter settings {valence, arousal} = [{0,0}; {0,0.5}; {0,1}] were used to evaluate the system's performance when valence is set to zero. The bar graphs depicting the averaged ratings (along with standard errors) are presented in Figure 1. As expected, a strong increasing trend is seen for both the average valence and arousal ratings with respect to their corresponding parameter settings. With regard to the valence ratings, we observe the majority of ratings to fall between the < 0.25 and > 0.75 parameter settings. It is common to see a higher density of responses in the middle of psychometric rating scales (e.g., with both ends of the scale receiving proportionally fewer responses; Leung, 2011). This could also indicate that the extremes of the valence parameter values are less distinguishable by listeners. On the other hand, a better correspondence is observed between average arousal ratings and the respective parameter values at all levels of arousal.

To test the relationship between average valence and arousal user ratings and parameter settings, we performed linear regression analyses (illustrated in Figure 1). The coefficient of determination is *R*^2^ = 0.90 (*F* = 27, *p* < 0.05) for valence, and *R*^2^ = 0.96 (*F* = 74, *p* < 0.01) for arousal, which confirms that both parameters are very effective in conveying their intended dimension of emotion. The results also show a stronger linear relationship for arousal (between average arousal ratings and parameter settings) in comparison to valence. This finding, in which arousal is more reliably expressed via music than valence, has previously been found in the literature (Wallis et al., 2011; Ehrlich et al., 2019). These results show that the music generated by AffectMachine-Classical generally conveys the intended levels of valence and arousal to listeners.

**Figure 2:**
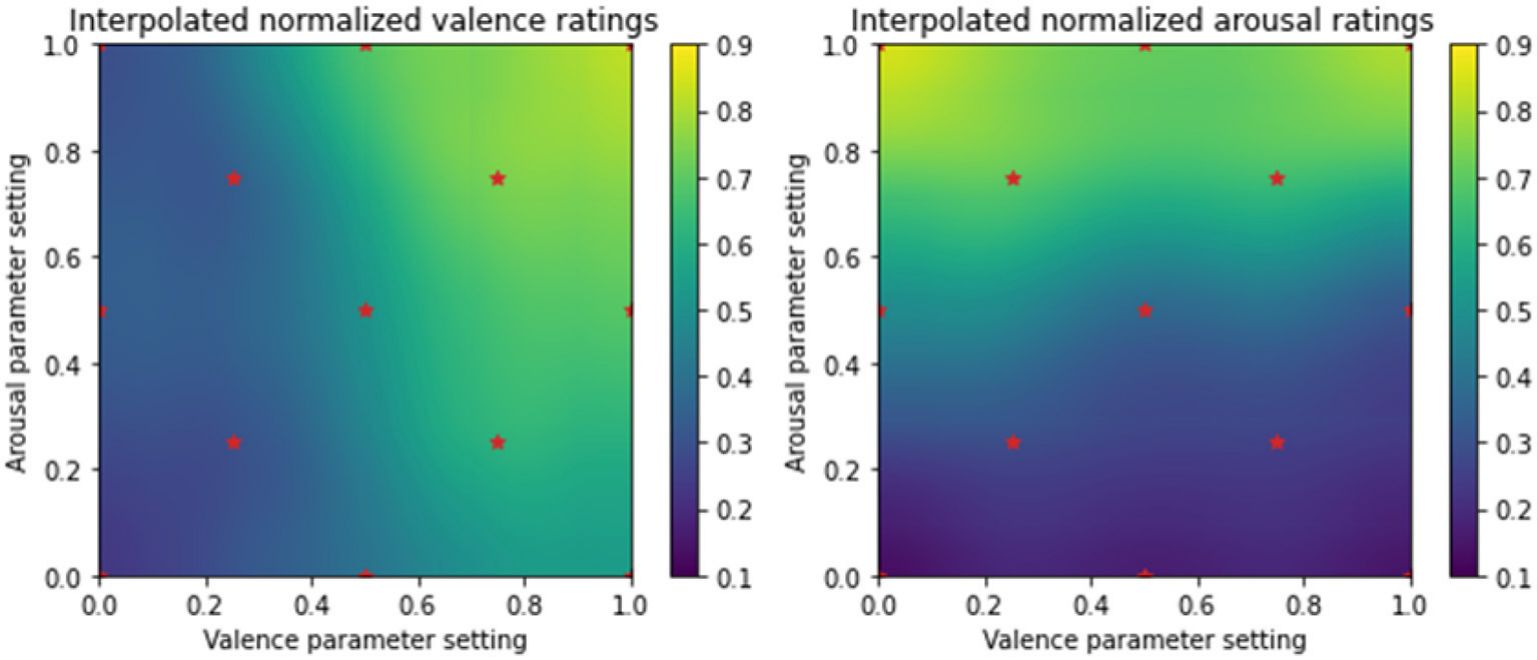
Average (interpolated) valence and arousal ratings as a function of the valence and arousal parameters. Vertical color bars represent the colors corresponding to different values of normalized average ratings over the range of 0.1–0.9.

Next, we investigate whether the perception of valence is influenced by changes in the arousal parameter setting, and conversely whether the perception of arousal is influenced by changes in the valence parameter setting. To do so, we analyse the dependence of average emotion ratings on both the valence and arousal parameter settings together. Figure 2 visualizes this dependence by presenting the interpolated average valence (left) and arousal ratings (right) as a function of the emotion parameter settings. The stars in the figure represent the 13 points around the valence and arousal plane used to generate musical stimuli. As can be seen in the figure on the left, the perceived valence is lower than the actual valence parameter setting (for *V* > 0.7) for excerpts expressing arousal values < 0.4. That is, excerpts generated to express high valence convey only moderate valence when the arousal setting is low. This may be due in part to the effect of a slower tempo. Ratings at low valence settings are, however, in accordance with their respective parameter values. In contrast, we observe uniform correspondence between the arousal parameter values and arousal ratings regardless of the valence parameter setting. Our study replicates a phenomenon that has been previously described in Wallis et al. (2011)—the authors found asymmetrical “crossover” effects between arousal and valence such that while perceived valence correlates with intended arousal, perceived arousal does not correlate significantly with intended valence.

To investigate these linear dependencies, we performed multiple linear regression between the valence and arousal parameter settings (independent variables) and average valence/arousal rating (dependent variable). The results indicate that perceived valence ratings are significantly influenced by both the valence (*F* = 63, *p* < 0.001) and arousal (*F* = 11, *p* < 0.01) parameter settings, which is in line with what we observed in Figure 2. Perceived arousal ratings, however, only show a significant dependence on the arousal settings (*F* = 153, *p* < 0.001). This observation is in line with findings from the literature which show that modeling the arousal component of emotion is more straightforward than the valence component (Yang et al., 2008; Wallis et al., 2011). Nevertheless, the obtained *R*^2^ values are high *R*^2^>0.85 for both average valence and arousal ratings. This confirms that irrespective of the emotion component, the majority of variability in average ratings during multiple regression analysis is explained by the valence and arousal settings values.

In summary, the listener study validates the ability of AffectMachine-Classical to generate music that expresses desired levels of emotion, measured in terms of arousal and valence. This confirms that the system has the potential to be deployed in applications that benefit from affective music—for example, the AffectMachine-Classical could be integrated with biofeedback systems wherein the music driven by the users' neural (or other physiological) signals can be used to reflect their emotional state. This direction is promising for developing more sophisticated emotion mediation systems with applications in healthcare (Agres et al., 2021). In the next section, we analyse the impact of participants' prior musical training on emotion ratings.

#### 3.2.2. Impact of prior musical training on emotion ratings

In this section, we present a comparison of user ratings provided by participants with and without prior musical training. Participants indicated whether they had prior musical training in the demographic questionnaire they completed. Based on participants' response to the question “Do you currently play an instrument (including voice)?” they were divided into two groups—the musical training (MT) group and no musical training (NMT) group. The MT and NMT groups have 12 and 14 participants, respectively.

**Figure 3:**
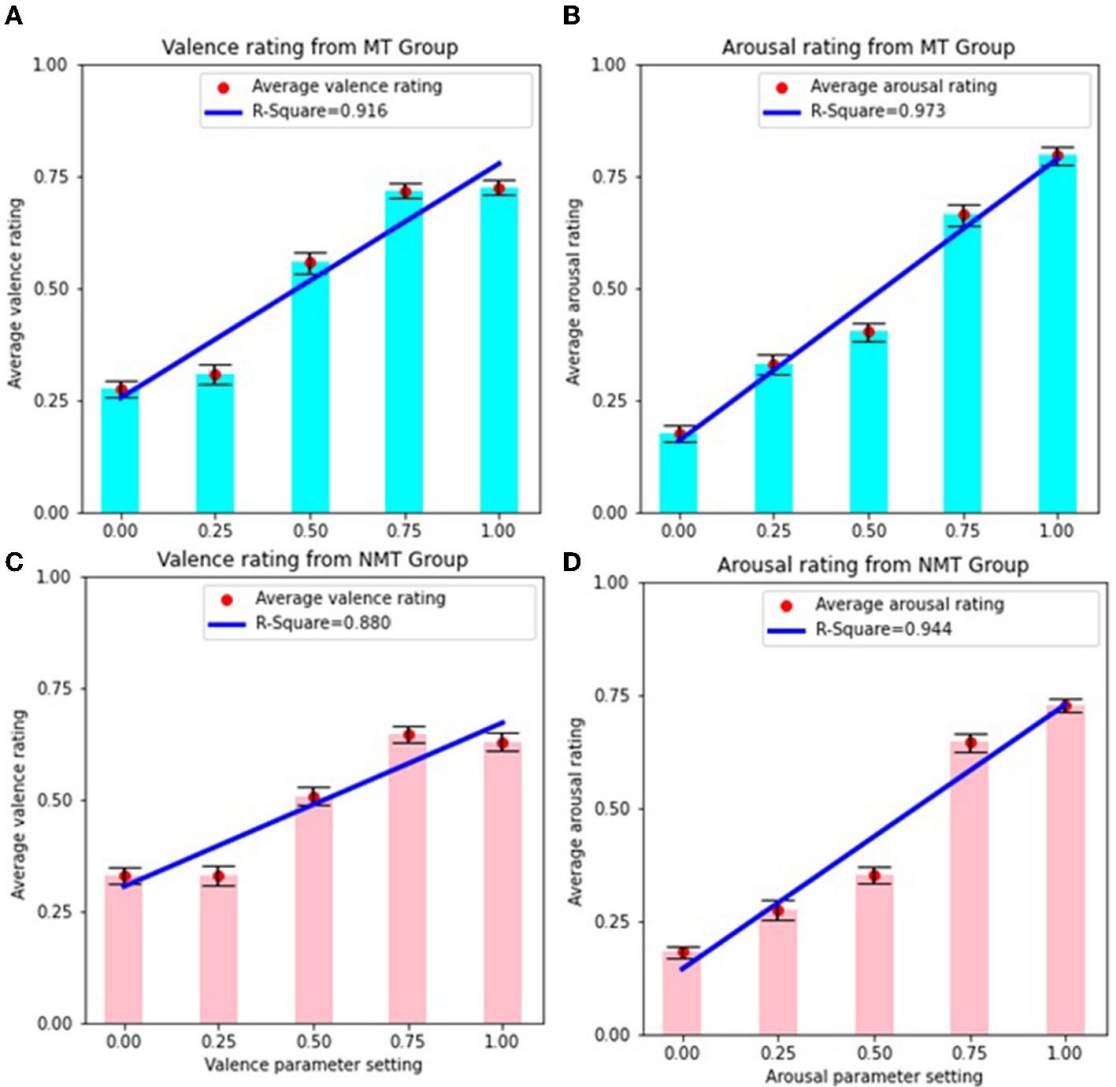
**(A)** Average valence rating and linear regression for MT group. **(B)** Average arousal rating and linear regression for MT group. **(C)** Average valence rating and linear regression for NMT group. **(D)** Average arousal rating and linear regression for NMT group. Error bars depict standard error.

Figure 3 presents the average emotion ratings corresponding to different levels of emotion parameter values for both the MT and NMT groups. As illustrated in the graphs, a stronger correspondence between the average emotion ratings and parameter-setting values is observed for arousal in comparison to valence, for both groups. As noted above, the average valence ratings demonstrate a saturation effect for lower (< 0.25) and higher (> 0.75) parameter-setting values for both the MT and NMT groups. Figure 3 also shows the linear regression fit for all the cases. The *R*^2^ values reflecting the relationship between emotion ratings and parameter settings are marginally higher for the MT group (*R*^2^ for valence is 0.91, *F* = 33, *p* = 0.01; *R*^2^ for arousal is 0.97, *F* = 111, *p* < 0.01) as compared with the NMT group (*R*^2^ for valence is 0.88, *F* = 22, *p* < 0.05; *R*^2^ for arousal is 0.94, *F* = 51, *p < 0.01*), for both valence and arousal. To compare whether the differences between these linear regression models were significant, we calculated the Akaike Information Criterion (AIC) for both the MT group (*AIC* = −10.65 for valence and *AIC* = −14.87 for arousal) and NMT group (*AIC* = −12.20 for valence and *AIC* = −11.74 for arousal). The statistics show that there is only a marginal difference (in perceived emotion ratings based on the system's emotion settings) between the MT and NMT groups. Although musical expertise has been found to influence the perception of emotion in affective music in some cases (e.g., see Di Mauro et al., 2018), we find here that both musicians and non-musicians reliably appraise the music created by AffectMachine-Classical as the emotion intended by the system. We note, however, that given the limited sample size in our study, it is difficult to generalize the effects of musical training, and a larger sample size could yield a significant difference between the two listener groups. Nevertheless, we observe that regardless of musical training, all of the participants were able to reliably perceive the emotional expression in the music, which is evident from the high *R*^2^ values observed (> 0.85) for both listener groups.

In addition, we also performed a multiple linear regression to examine the effect of parameter settings in both emotion dimensions on individual perceived emotion ratings. We obtained high *R*^2^ values (*R*^2^ > 0.8) for all the scenarios, i.e., for both emotion dimensions for both groups. Furthermore, we observed that perceived valence is significantly influenced by both valence (*F* = 89, *p* < 0.001 for MT, and *F* = 38, *p* < 0.001 for NMT) and arousal (*F* = 5.8, *p* < 0.05 for MT, and *F* = 15, *p* < 0.01 for NMT) parameter settings for both MT and NMT groups. However, perceived arousal ratings are only influenced by the arousal settings (*F* = 216, *p* < 0.001 for MT and *F* = 110, *p* < 0.001 for NMT), and not valence settings, in both groups. These findings are similar to what we observed for all the participants without any grouping.

## 4. General discussion

In this paper, we present a new computational system for generating affective classical music called AffectMachine-Classical. The system provides a probabilistic, rule-based algorithm for flexibly generating affective music in real-time. AffectMachine's behavior essentially resembles semi-structured musical improvisation, not dissimilar to the approach utilized by Baroque composers/musicians (Moersch, 2009), or how human jazz performers might follow the basic melody outlined by a lead sheet while coming up with reharmonizations, chord voicings, and appropriate accompaniments, on the fly, to help convey the emotions they are aiming to express (Johnson-Laird, 2002; McPherson et al., 2014). To our knowledge, ours is the first affective music generation system to adopt this approach. A key advantage of this method is that the music generated by the system achieves a balance between musical coherence and self-similarity, which may be valuable in research and other contexts that require lengthier pieces of music. Although the issue of artificially generating music that is capable of exhibiting long-term structure has been described as “notoriously difficult” (Carnovalini and Rodà, 2020) and cited as one of the grand challenges for automatic music generation (Herremans et al., 2017; Briot and Pachet, 2020), our system addresses this issue in part by providing a structural frame by means of an 8-bar form in which the music is generated. Melodic coherence is maintained due to the constraints enforced by the algorithms used to generate melodic patterns, and harmonic coherence is achieved through the use of a chord matrix based on the 8-bar form.

AffectMachine was developed to be embedded into real-time biofeedback systems, such as music-based Brain-Computer Interfaces (BCIs), to leverage neurofeedback and adaptive, affective music generation to help the listener achieve a target emotion state. The listener study reported here was conducted to validate the efficacy of the system for generating affective music. Indeed, regardless of musical experience, listeners perceived the target emotion of the musical excerpts (in terms of arousal and valence), as intended by the system.

The results of the listener study indicate a strong relationship between the arousal parameter setting and average arousal ratings (*R*^2^ = 0.96), as well as the valence setting and average valence ratings (*R*^2^ = 0.90). The correlation between target and perceived emotion was more tempered for valence compared to arousal, as previously found in the literature (e.g., Wallis et al., 2011; Ehrlich et al., 2019). From the results of our listener study, it is evident that AffectMachine is capable of expressing the desired emotional information, and thus holds the potential to be used as an affect guide for mediating/regulating the emotion states of listeners. We would like to emphasize that despite the differences in listeners' prior musical training, individual and cultural preferences, and demographic profile, there was strong evidence suggesting that the system's target emotions were indeed perceived as intended across listeners, which makes Affect Machine-Classical a very promising tool for creating music with reliable emotion perception.

In terms of future directions, as discussed above, AffectMachine will be embedded into biofeedback systems, such as a Brain-Computer-Interface (similar to Ehrlich et al., 2019), to support emotion self-regulation in listeners. Further, our system may be used for wellness applications such as generating affective music “playlists” for emotion mediation. That is, using the flexible music generation system, a user may pre-define an “emotion trajectory” (e.g., a path through emotion space, such as the two-dimensional Valence-Arousal space) to define the emotional qualities of their music over the duration of listening. For example, if a user desires 10 min of music to help him move from a depressed emotion state to a happy emotion state, he may indicate an emotion trajectory from negative arousal/valence to positive arousal/valence over the specified duration, and the system will create bespoke affective music to this specification. Therefore, AffectMachine has the potential to be embedded in various kinds of well-being applications to create highly-personalized, affective music for emotion regulation.

## Data availability statement

The raw data supporting the conclusions of this article will be made available by the authors, without undue reservation.

## Ethics statement

The studies involving human participants were reviewed and approved by the Institutional Review Board (IRB) of the National University of Singapore (NUS). The patients/participants provided their written informed consent to participate in this study.

## Author contributions

KA and AD led the research. KA initiated, supervised the project, and led the manuscript preparation and revision. AD led data collection for the listener study and as well as data analysis and reporting. PC led the system development, under the supervision of KA, and as well as the system description. All authors contributed to writing the paper and approved of the submitted version.

## Footnotes

^1^Statistics from the National Institute of Mental Health: https://www.nimh.nih.gov/health/statistics/mental-illness.

^2^ https://openai.com/

^3^A separate version of AffectMachine is being developed and tested in a popular-music genre, to afford listeners some variety and choice based on their musical preferences.
